# Examining the relationship between lifetime stressful life events and the onset of major depression in Chinese women^☆^

**DOI:** 10.1016/j.jad.2011.06.054

**Published:** 2011-12

**Authors:** Ming Tao, Yihan Li, Dong Xie, Zhiyang Wang, Jianying Qiu, Wenyuan Wu, Jing Sun, Zhoubing Wang, Danhong Tao, Hongsu Zhao, Tian Tian, Jingxuan Zhang, Chengge Gao, Qihui Niu, Qiang Li, Shanming Liu, Jia Liu, Yunshu Zhang, Qiang He, Han Rong, Zhaoyu Gan, Jianying Li, Xiansheng Chen, Jiyang Pan, Yi Li, Yanping Cui, Wei Han, Huan Ma, Shoufu Xie, Guixing Jin, Ling Li, Ruiling Zhang, Qingrong Tan, Jun Zhang, Jing Guan, Shenxun Shi, Yiping Chen, Kenneth S. Kendler, Jonathan Flint, Jingfang Gao

**Affiliations:** a Second Affiliated Hospital of Zhejiang Chinese Medical University, No. 318 Chao Wang Road, Hangzhou, Zhejiang 310005, PR China; b Wellcome Trust Centre for Human Genetics, Oxford OX3 7BN, UK; c Fudan University Affiliated Huashan Hospital, No. 12 Wulumuqi Zhong Road, Shanghai, 200040, PR China; d Shanghai Jiao Tong University School of Medicine Affiliated Shanghai Mental Health Centre, No. 600 Wan Ping Nan Road, Shanghai 200030, PR China; e Shanghai Tongji University Affiliated Tongji Hospital, No. 389 Xinchun Road, Shanghai 200065, PR China; f Nanjing Brain Hospital, No. 264 Guangzhou Road, Nanjing, Jiangsu, 210029, PR China; g No. 4 Affiliated Hospital of Jiangsu University, No. 246 Nan Men Da Street, Zhenjiang, Jiangsu, 212001, PR China; h Zhejiang Traditional Chinese Medical Hospital, No. 54 You Dian Road, Hangzhou Zhejiang 310006, PR China; i Tianjin Anding Hospital, No. 13 Liu Lin Road, Hexi District, Tianjin, 300222, PR China; j Shandong Mental Health Center, No. 49 East Wenhua Road, Jinan, Shandong 250014, PR China; k No. 1 Hospital of Medical College of Xian Jiaotong University, No. 277 West Yan Ta Road, Xi'an, Shaanxi, 710061, PR China; l No. 1 Hospital of Zhengzhou University, No. 1 East Jianshe Road, Zhengzhou, Henan 450052, PR China; m No. 1 Mental Health Center Affiliated Harbin Medical University, No 23 You Zheng Jie, Nangang District, Harbin, Heilongjiang, PR China; n Mental Health Center of West China Hospital of Sichuan University, No. 28 Dian Xin Nan Jie, Wu Hou District, Chengdu, Sichuan 610041, PR China; o Beijing Anding Hospital, Capital Medical University, No. 5 An Kang Hutong Deshengmen Wai, Xicheng District, Beijing 100088, PR China; p Hebei Mental Health Center, No. 572 Dongfeng Road, Baoding, Hebei 071000, PR China; q Shengjing Hospital of China Medical University, No. 36 Sanhao Street, Heping District Shenyang, Liaoning, 110817, PR China; r Shenzhen Kangning Hospital, No. 1080, Cui Zu Street, Luo Hu, Shenzhen, 518020, PR China; s No. 3 Affiliated Hospital of Sun Yat-sen University, No. 600 Tian He Road, Tian He District, Guangzhou, Guangdong, 510630, PR China; t No. 1 Hospital of Shanxi Medical University, No. 85 Jiefang South Road, Taiyuan, Shanxi, 030001, PR China; u Mental Hospital of Jiangxi Province, No. 43 Shangfang Road, Nanchang, Jiangxi, 330029, PR China; v The First Affiliated Hospital of Jinan University, No. 613 West Huangpu Avenue, Guangzhou, 510630, PR China; w Wuhan Mental Health Center, No. 70, You Yi Road, Wuhan, 430022, PR China; x No. 3 Hospital of Heilongjiang Province, No. 135 Jiao Tong Lu, Beian, Heilongjiang, PR China; y Jilin Brain Hospital, No. 98 Zhong Yang Xi Lu, Siping, Jilin, 136000, PR China; z The First Hospital of China Medical University, No. 155 Nanjing Bei Jie, He Ping District, Shenyang, 110001, PR China; aa Dalian No. 7 People's Hospital & Dalian Mental Health Center, No. 179 Ling Shui Lu, Gan Jing Zi District, Dalian, PR China; ab The First Hospital of Hebei Medical University, No. 89 Donggang Road, Shijiazhuang, 050031, PR China; ac Lanzhou University Second Hospital, Second Clinical Medical College of Lanzhou University, No. 82, Cui Ying Men, Lanzhou, Gansu, 730030, PR China; ad Psychiatric Hospital of Henan Province, No. 388 Jian She Zhong Lu, Xinxiang, Henan, PR China; ae The Fourth Military Medical University Affiliated Xijing Hospital, No. 17, Changle West Road, Xi'an, Shaanxi, 710032, PR China; af No. 4 People's Hospital of Liaocheng, No. 47 Hua Yuan Bei Road, Liaocheng, Shandong, 252000, PR China; ag Guangzhou Brain Hospital/Guangzhou Psychiatric Hospital, No. 36 Ming Xin Lu, Fang Cun Da Dao, Li Wan District, Guangzhou 510370, PR China; ah Clinical Trial Service Unit, Richard Doll Building, Old Road Campus, Roosevelt Drive, Oxford, OX3 7LF, UK; ai Virginia Commonwealth University, Department of Psychiatry, Virginia Institute for Psychiatric and Behavioral Genetics, Richmond, VA 23298-0126, USA

**Keywords:** Major depressive disorder, Stressful life event, Social adversity, Symptom

## Abstract

**Background:**

In European and US studies, patients with major depressive disorder (MDD) report more stressful life events (SLEs) than controls, but this relationship has rarely been studied in Chinese populations.

**Methods:**

Sixteen lifetime SLEs were assessed at interview in two groups of Han Chinese women: 1970 clinically ascertained with recurrent MDD and 2597 matched controls. Diagnostic and other risk factor information was assessed at personal interview. Odds ratios (ORs) were calculated by logistic regression.

**Results:**

60% of controls and 72% of cases reported at least one lifetime SLE. Fourteen of the sixteen SLEs occurred significantly more frequently in those with MDD (median odds ratio of 1.6). The three SLEs most strongly associated with risk for MDD (OR > 3.0) preceded the onset of MDD the majority of the time: rape (82%), physical abuse (100%) and serious neglect (99%).

**Limitations:**

Our results may apply to females only. SLEs were rated retrospectively and are subject to biases in recollection. We did not assess contextual information for each life event.

**Conclusions:**

More severe SLEs are more strongly associated with MDD. These results support the involvement of psychosocial adversity in the etiology of MDD in China.

## 1 Introduction

A large body of evidence indicates that patients with major depressive disorder (MDD) report more stressful life events (SLEs) than controls (Hammen, 2005; Kessler, 1997; Paykel, 2003), but these studies have been almost entirely carried out in European and US populations. Given the importance attributed to life events as a potential causal factor in MDD and the admitted difficulties in demonstrating causality (Andrews and Tennant, 1978; Brown and Harris, 1978; Kendler et al., 1999, 2003; Paykel, 1978; Surtees et al., 1986), the dearth of work on non-Western populations is surprising.

The strength and the nature of the association between SLEs and MDD depend considerably on the characteristics of those exposed and the environment in which the event occurs (Kessler, 1997). MDD can be the cause, as well as the consequence, of some SLEs (for example depression in one partner may precipitate the break-up of a relationship); SLEs have a large effect on genetically predisposed individuals, as indexed for example by higher rates of neuroticism (Foley et al., 1996; Kendler et al., 1993; Lyons et al., 1993; McGue and Lykken, 1992). Such reciprocal relationships make it extremely challenging to determine how and when SLEs operate.

One unexplored avenue is investigating whether the same SLEs operate equivalently in different environments. Taking advantage of the remarkable cultural diversity of human populations and asking to what extent SLEs vary in their impact on MDD might identify stress-modifiers in the environment and thereby illuminate the stress-depression diathesis. We addressed this issue by asking whether SLEs associated with MDD in European and US populations have an equivalent relationship in an East Asian population. We examined 1970 Chinese women with recurrent MDD, and compared the prevalence of 16 SLEs in matched controls.

## 2 Method

### 2.1 Subjects

Data for the present study draw upon the ongoing China, Oxford and VCU Experimental Research on Genetic Epidemiology (CONVERGE) study of MDD. These analyses were based on a total of 1970 cases recruited from 53 provincial mental health centers and psychiatric departments of general medical hospitals in 41 cities in 19 provinces and four central cities Beijing, Shanghai, Tianjin and Chongqing, and 2597 controls who were recruited from patients undergoing minor surgical procedures at general hospitals or from local community centers.

All cases and controls were female and had four Han Chinese grandparents. Cases and controls were excluded if they had a pre-existing history of bipolar disorder, any type of psychosis or mental retardation. Cases were aged between 30 and 60, had two or more episodes of MDD, with the first episode occurring between 14 and 50 and had not abused drug or alcohol before the first episode of MDD. Controls were chosen to match the region of origin of cases, were aged between 40 and 60, had never experienced an episode of MDD and were not blood relatives of cases. An older minimal age of controls was used to reduce the chances that they might have a subsequent first onset of MDD. The mean age (and SD) of cases and controls in the dataset was respectively 45.1 (8.8) and 47.7 (5.5).

All subjects were interviewed using a computerized assessment system, which lasted an average of 2 h for a case and 1 h for a control. All interviewers were trained by the CONVERGE team for a minimum of 1 week in the use of the interview. The interview includes assessment of psychopathology, demographic and personal characteristics, and psychosocial functioning. Interviews were tape-recorded and a proportion of them were listened to by the trained editors who provided feedback on the quality of the interviews.

The study protocol was approved centrally by the Ethical Review Board of Oxford University and the ethics committee in participating hospitals in China.

### 2.2 Measures

The diagnoses of depressive (dysthymia and major depressive disorder) and anxiety disorders (generalized anxiety disorder, panic disorder with or without agoraphobia) were established with the Composite International Diagnostic Interview (CIDI) (WHO lifetime version 2.1; Chinese version), which classifies diagnoses according to the Diagnostic and Statistical Manual of Mental Disorders (DSM-IV) criteria (American Psychiatric Association, 1987). The interview was originally translated into Mandarin by a team of psychiatrists in Shanghai Mental Health Centre, with the translation reviewed and modified by members of the CONVERGE team. Phobias, divided into five subtypes (animal, situational, social and blood-injury, and agoraphobia), were diagnosed using an adaptation of the DSM-III criteria requiring one or more unreasonable fears, including fears of different animals, social phobia and agoraphobia, that objectively interfered with the respondent's life. The section on the assessment of phobias was translated by the CONVERGE team from the interview used in the Virginia Adult Twin Study of Psychiatric and Substance Use Disorders (VATSPUD) (Kendler and Prescott, 2006).

Additional information using instruments employed from VATSPSUD, translated and reviewed for accuracy by members of the CONVERGE team, was collected on premenstrual syndrome, postnatal depression, parent child relationship, stressful life events, social life, childhood sexual abuse, smoking and neuroticism etc. The stressful life events section, also developed for the VATSPSUD study, assessed 16 traumatic lifetime events and the age at their occurrence. The childhood sexual abuse was a shortened version of the detailed module used in the VATSPSUD study (Zlotnick et al., 2000).

Both the case and control interviews were fully computerized into a bilingual system of Mandarin and English developed in house in Oxford, and called SysQ. Skip patterns were built into SysQ. Interviews were administered by trained interviewers and entered offline in real time onto SysQ, which was installed in the laptops. Once an interview was completed, a backup file containing all the previously entered interview data could be generated with database compatible format. The backup files together with an audio recording of the entire interview were uploaded to a designated server currently maintained in Beijing by a service provider. All the uploaded files in the Beijing server were then transferred to an Oxford server quarterly.

### 2.3 Statistical analysis

Analyses were carried out by SPSS version 17.0 (SPSS Inc., Chicago, IL). Linear and logistic regression models were used to determine the independent association of SLEs with phenotypes. Coefficient values, odds ratios and 95% confidence intervals were used to quantify the strength of associations. In all analyses we included educational status and occupation as covariates, since SLEs may occur more frequently with lower socio-economic status.

## 3 Results

We obtained life events data on 1639 cases of recurrent MDD and in 2283 controls. Of these, 72% of cases and 59% of controls reported the occurrence of one or more SLEs over their lifetime. Table 1 shows that significantly more cases experience multiple SLEs than controls. We estimated the effect of number of life events on the risk of MDD by logistic regression and obtained a highly significant OR of 1.24 (95% confidence intervals 1.19–1.29, P = 6.49E-25). We see an increase in the OR for the prediction of MDD with an increasing number of SLEs. For those experiencing five or more SLEs the OR for MDD is 1.92 (95% confidence intervals, 1.09–3.36, P = 0.02).

We next considered the impact of each SLE individually. For each SLE, we estimated the odds ratio for increasing the risk of MDD by logistic regression and show the results in Table 2. Fourteen of the sixteen SLEs are significantly associated with MDD, with a skewed distribution of ORs (median 1.6). Notably we find that stressful life events involving personal assault (physical abuse, physical assault and rape) have the highest ORs, with the highest (> 11) associated with a particularly severe event: rape.

Table 3 shows the information we obtained on the relationship between each category of SLE and the first episode of MDD. We report the fraction of cases reporting the SLE preceding the onset of MDD, and the fraction reporting that the SLE occurred prior to the onset of MDD. The 16 SLEs examined in this paper fell into 3 groups. For six (natural disaster, rape, physical assault, physical abuse, serious neglect and other terrible event), a very large preponderance of the time (> 80%), the SLE preceded the first onset of MDD. For five of the SLEs (financial crisis, serious illness, life-threatening accident, witness someone injured, and being threatened), the SLE preceded the first onset of MDD 70–79% of the time. For the remaining five events, the SLE preceded the first onset of MDD 50–69% of the time.

## 4 Discussion

We found that Chinese women with recurrent MDD report a significantly higher prevalence of lifetime SLEs compared to controls. Our results are important because they largely replicate findings in Western populations.

First, in agreement with Western reports, we find that SLEs are common (Bidzinska, 1984; Billings et al., 1983; Holahan and Moos, 1991; Kendler et al., 1993; Shrout et al., 1989; Williamson et al., 1995). Over half of our controls reported at least one such event and almost three quarters of our cases. Though this is less than the doubling of events reported in reviews of case control studies (Mazure, 1998), it implies that, as in Western populations, in China only a minority of those exposed to an SLE will go on to develop MDD (Kessler, 1997).

Second, we have evidence for a dose–response relationship between MDD and SLEs. We find that MDD patients are more likely to have experienced more SLEs than controls, and we have some evidence that more severe SLEs are more strongly associated with MDD. We find that rape has a much larger effect than the other SLEs. In Western studies undesirable, major SLEs are more likely to be associated with depressive onset (Grant et al., 1976).

Third, consistent with the hypothesis that the association between the observed SLEs and MDD results largely from a causal impact of SLEs on risk for MDD, all three of the SLEs especially strongly associated with risk for MDD (that is to say with OR > 3.0) preceded the onset of MDD the great majority of the time: rape (82%), physical abuse (100%) and serious neglect (99%) (Table 3). Our results also replicate prior studies that childhood adversities, especially physical abuse, and serious neglect substantially increase risk for MDD (Green et al., 2010). Thus at least some SLEs precede the occurrence of MDD, consistent with their having a causal role.

While our evidence suggests a causal relationship between SLEs and MDD it is also consistent with other interpretations that have also emerged from Western studies of causal inference. For example, an analysis of SLEs that the individual has contributed to producing, termed dependent SLEs, shows both causal and non-causal associations with MDD (Kendler and Gardner, 2010). This is not to say dependent SLEs are a relatively less important risk factor for MDD. In fact the opposite is true: Hammen reported that, compared to controls, women with recurrent MDD were more likely to experience high levels of dependent SLEs (Hammen, 1991) and dependent SLEs are more predictive of depression onset than independent events (Kendler et al., 1999). It indicates rather the SLEs and MDD may have a more complex relationship than is at first apparent.

Stress can be the outcome of depression, or both can emerge from a moderating factor, such as personality (Kendler et al., 2004). The existence of a bidirectional relationship between stress and depression has generated considerable interest (Rutter, 1986). This relationship in part has a biological origin: it is now known that genetic factors influence exposure to SLEs (Foley et al., 1996; Kendler et al., 1993), and that there exist genetic variants which have two effects: they increase vulnerability to MDD and increase an individuals' probability of selecting themselves into a high-risk environment, where they are more likely to experience SLEs (Kendler et al., 1999, 2004; Kendler and Karkowski-Shuman, 1997).

Our results should be interpreted in the context of four potential methodological limitations. First, the sample is entirely female and it cannot be assumed that the same relationship between SLEs and MDD would be seen in males. Second, since both SLEs and the onset of MDD were rated retrospectively, our data are subject to biases in recollection. However, it is unlikely that our depressed patients had a general tendency to report high rates of all prior stressors, as several of our SLEs occurred no more frequently in our cases than in our matched controls. Third, we did not assess contextual information for each life event: we did not judge severity or “degree of threat”. Fourth, our main analyses could not discriminate the degree to which the observed associations between SLEs and MDD were a result of SLE exposure increasing risk for MDD or MDD increasing the risk for SLEs. Partial information to address this question was available to us as we recorded the age at which each SLE was first experienced and the age at onset of MDD. SLEs that preceded the first onset of MDD logically could not be a result of MDD. SLEs that followed the first episode of MDD could be a result of prior depressive episodes or a cause of subsequent episodes.

Research into the impact of SLEs has begun to consider the environment in which they occur, the nature of the person who experiences them and the interaction of the two (Kessler, 1997). Studies taking these factors into account demonstrate the difficulties facing attempts to establish the causal role of SLEs on MDD. Currently our data are not able to address these issues. We do not for example rate the context in which SLEs occur and we have not distinguished between dependent and independent events (ratings of event dependence reflect the likelihood that an SLE could be due to the subject's own behavior). However the similarity of our findings to those obtained in Europe and the US suggests that the nature and action of SLEs may be relatively invariant to cultural context. More detailed analysis, taking into account genetic variation, may allow us to pick apart the pathway from stressor to illness.

## Role of funding source

Funding for this study was provided by the Wellcome Trust; the Wellcome Trust had no further role in study design; in the collection, analysis and interpretation of data; in the writing of the report; and in the decision to submit the paper for publication.

## Conflict of interest

All authors declare they have no conflicts of interest including any financial, personal or other relationships with other people or organizations within 3 years of beginning the work submitted that could inappropriately influence, or be perceived to influence, their work

## Figures and Tables

**Table 1:** Frequency of stressful life events.

	Cases		Controls	
Number of SLEs	N	%	N	%
0	459	28.00%	935	41.00%
1	449	27.40%	643	28.20%
2	325	19.80%	384	16.80%
3	174	10.60%	181	7.90%
4	116	7.10%	87	3.80%
5	61	3.70%	31	1.40%
6	23	1.40%	18	0.80%
7	18	1.10%	2	0.10%
> 7	14	0.90%	2	0.10%

The number (N) and percentage (%) of stressful life events (SLEs) in cases of recurrent major depression and controls. The difference between the two groups is highly significant: *χ*^2^ = 136.3, df = 8, p-value < 2.2e-16.

**Table 2:** The impact on major depressive disorder of 16 stressful life events in Chinese women with recurrent major depression.

Stressful life events	Cases		Controls		OR	95% CI	LogP
	N	Pct	N	Pct			
Death of a family member	403	20.7%	442	17.1%	1.27	1.09–1.47	2.73
Divorce/relationship breakup	340	17.5%	222	8.6%	2.26	1.88–2.70	18.00
Ever unemployed	319	16.4%	348	13.4%	1.26	1.07–1.49	2.26
Job loss	144	7.4%	98	3.8%	2.03	1.56–2.64	6.86
Financial crisis	420	21.6%	448	17.3%	1.32	1.13–1.53	3.53
Legal problems	81	4.2%	38	1.5%	2.92	1.97–4.30	7.12
Serious illness	232	11.9%	243	9.4%	1.31	1.08–1.58	2.23
Life-threatening accident	188	9.7%	207	8.0%	1.23	1.00–1.52	1.31
Natural disaster	264	13.6%	379	14.6%	0.92	0.77–1.08	0.52
Witness someone injured	174	8.9%	233	9.0%	0.99	0.81–1.22	0.02
Raped	33	1.7%	4	0.2%	11.15	3.94–31.51	5.26
Physically assaulted	122	6.3%	89	3.4%	1.88	1.42–2.49	5.00
Physically abused	103	5.3%	42	1.6%	3.39	2.36–4.88	10.31
Seriously neglected	203	10.5%	74	2.9%	3.98	3.03–5.23	22.43
Threatened	26	1.3%	14	0.5%	2.50	1.30–4.80	2.23
Other terrible event	154	8.0%	142	5.5%	1.49	1.18–1.88	3.02

The number (N) and percentage (Pct) of women reporting each of 16 stressful life events are given. Odds ratio (OR) with their 95% confidence intervals (95% CI) and associated P-values are shown. The P-value (LogP) is given as the negative logarithm (base 10).

**Table 3:** Occurrence of stressful life events in relation to MDD onset.

	SLE before MD onset		SLE after MD onset	
	N	Pct	N	Pct
Death of a family member	200	10.3%	168	8.6%
Divorce/relationship breakup	184	9.5%	136	7.0%
Ever unemployed	191	9.8%	99	5.1%
Job loss	77	4.0%	56	2.9%
Financial crisis	274	14.1%	112	5.8%
Legal problems	36	1.8%	38	2.0%
Serious illness	156	8.0%	58	3.0%
Life-threatening accident	136	7.0%	40	2.1%
Natural disaster	198	10.2%	50	2.6%
Witness someone injured	109	5.6%	50	2.6%
Raped	27	1.4%	5	0.3%
Physically assaulted	89	4.6%	23	1.2%
Physically abused	96	4.9%	0	0.0%
Seriously neglected	188	9.7%	1	0.1%
Threatened	18	0.9%	7	0.4%
Other terrible event	117	6.0%	27	1.4%

The number (N) and percentage (Pct) of stressful live events that were reported to occur before the first episode of major depressive disorder (MDD).
